# The Prognostic Role of C‐Reactive Protein–Triglyceride Glucose Index in Predicting Unfavorable Outcomes in Acute Ischemic Stroke: A Large‐Scale Cohort Study

**DOI:** 10.1002/brb3.71578

**Published:** 2026-07-09

**Authors:** Huang Luwen, Chen Qin, Xu Lei, Yu Ming, Li Linlin

**Affiliations:** ^1^ Department of Neurology Suining Central Hospital Suining China

**Keywords:** acute ischemic stroke, CTI, inflammation, insulin resistance, unfavorable outcome

## Abstract

**Background:**

The C‐reactive protein–triglyceride glucose index (CTI) has been established as a novel biomarker reflecting insulin resistance and systemic inflammation. However, its association with unfavorable outcomes in acute ischemic stroke (AIS), especially when patients are stratified by glycemic status, remains unclear.

**Methods:**

A total of 1485 patients with AIS admitted to Seoul National University Hospital between 2010 and 2016 were included in this study. The primary outcome was poor prognosis, defined as a modified Rankin scale score ≥3 at 3 months. The CTI was calculated via the following formula: 0.412 × ln (hs‐CRP [mg/L]) + ln (TG [mg/dL] × FBG [mg/dL])/2. Logistic regression models and restricted cubic spline analyses were used to evaluate the associations between the CTI and poor stroke outcomes, with stratification by sex and glycemic status. Subgroup analyses and propensity score analyses were also conducted to validate the robustness of the findings.

**Results:**

At 3 months after AIS onset, 414 patients (27.88%) experienced poor outcomes. Our findings revealed a significant positive linear association between CTI levels and the risk of unfavorable outcomes in AIS patients. The association was significant in both sexes, with a higher odds ratio observed in males (OR 1.591, 95% CI: 1.235–2.050) than in females (OR 1.347, 95% CI: 1.004–1.807). When individuals were stratified by glycemic status, an elevated CTI was significantly associated with an increased risk of poor outcomes among individuals with prediabetes (pre‐DM) (OR 1.634, 95% CI: 1.267–2.108) and diabetes mellitus (DM) (OR 1.819, 95% CI: 1.315–2.517), whereas no statistically significant association was observed in participants with normal glucose regulation.

**Conclusions:**

Elevated CTI levels were significantly associated with an increased risk of unfavorable outcomes in AIS patients, with a stronger association observed in males. This relationship remained significant among individuals with prediabetes and diabetes but was not evident in those with normal glucose regulation. These findings suggest that the CTI may serve as a simple and effective biomarker for identifying AIS patients at greater risk of poor prognosis.

## 1 Introduction

Stroke is a leading cause of death and disability worldwide, with acute ischemic stroke (AIS) accounting for 85% of all strokes (GBD 2016 Stroke Collaborators 2019; eClinicalMedicine 2023; GBD 2016 Neurology Collaborators 2019; Fatahzadeh and Glick 2006; Ding et al. 2022). AIS is characterized by stenosis or occlusion of arteries supplying blood to the brain, resulting in brain tissue necrosis and inadequate cerebral perfusion (Feske 2021). From 1990–2019, the incidence of AIS significantly increased worldwide, with an overall increase of 87.55% (Ding et al. 2022). According to the 2019 Global Burden of Disease study, there were 7,630,800 ischemic stroke events globally (Ding et al. 2022). During this 30‐year period, the number of deaths associated with AIS increased by 60.68%, and the disability‐adjusted life years corresponding to these deaths increased by 56.72% (Ding et al. 2022). Therefore, identifying effective risk factors for predicting poor outcomes is essential for improving patient management and guiding therapeutic interventions.

The triglyceride‐glucose (TyG) index, first proposed in 2008, is a reliable biomarker of insulin resistance and has been widely used in clinical practice (Simental‐Mendía et al. 2008). Increasing evidence suggests a significant correlation between the TyG index and poor outcomes in AIS patients (Yang et al. 2023; Zhou et al. 2020). In addition, inflammation is recognized as a critical risk factor for unfavorable outcomes in AIS patients (X. Liu et al. 2021; Jin et al. 2023; Zhou et al. 2022). High‐sensitivity C‐reactive protein (hs‐CRP), a nonspecific inflammatory marker, is closely associated with poor outcomes in AIS patients and is considered a promising biomarker for AIS prognosis assessment (F. Liu et al. 2023; Munir et al. 2021). Therefore, the development of a composite index that reflects both insulin resistance and inflammation to predict poor outcomes in AIS patients is highly important. The C‐reactive protein‐triglyceride glucose index (CTI), derived from CRP and TyG, combines these two key indicators (Ruan et al. 2022). Initially, proposed to predict poor cancer prognosis (Ruan et al. 2022), the CTI has since demonstrated significant predictive value for various conditions, including adult depression (Huang et al. 2024), endometriosis (Ren et al. 2025), male erectile dysfunction (Mei et al. 2024), and stroke incidence across diverse populations (Tang et al. 2024; Huo et al. 2025).

Currently, there is a lack of research on the association between the CTI and AIS prognosis, and the role of the CTI in predicting poor outcomes in AIS patients across different glucose metabolic states remains unclear. Therefore, this study aimed to investigate the complex relationship between the CTI and AIS prognosis in individuals with varying glucose metabolic statuses, providing a more accurate biomarker for clinical application.

## 2 Methods

### 2.1 Study Design and Participants

**FIGURE 1:**
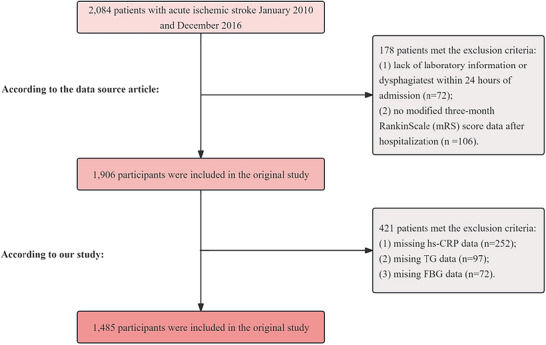
Flow chart of the study population.

The study utilized data collected from January 2010 to December 2016 at Seoul National University Hospital (Kang et al. 2020). The initial trial included 2084 individuals with AIS who were admitted within 7 days of the onset of symptoms on the basis of a prospective registry approach. The exclusion criteria were as follows: (1) absence of laboratory data or dysphagia assessment within 24 h of admission (*n* = 72) and (2) lack of 3‐month modified Rankin scale (mRS) score data posthospitalization (*n* = 106). Note that 1906 patients remained for baseline evaluation. Additionally, our study revealed the following: (1) missing hs‐CRP data (*n* = 252), (2) missing TG data (*n* = 97), (3) missing FBG data (*n* = 72), (4) missing WBC data (*n* = 1), (5) missing stroke etiology data (*n* = 1), and (6) missing mRS data at admission (*n* = 1). A total of 1485 patients were included in the final analysis (Figure 1).

This study was approved by the institutional review board of Seoul National University Hospital (IRB No. 1009‐062‐332), with a waiver of patient consent (Kang et al. 2020).

### 2.2 Calculation of the CTI

The CTI index was calculated via the following formula: CTI  =  0.412 × Ln (CRP [mg/L]) + Ln (TG [mg/dL] × FBG [mg/dL])/2.

### 2.3 Assessment of 3‐Month Unfavorable Outcomes in AIS Patients

The 3‐month prognosis of AIS patients was assessed via the mRS (Le Bouc et al. 2018). Data were collected from outpatient records and telephone interviews conducted 3 months post‐AIS (Kang et al. 2020). An mRS score of 3 or greater was classified as an unfavorable outcome, whereas a score of 2 or lower was considered a favorable outcome (Le Bouc et al. 2018).

### 2.4 Assessments of Covariates

The covariates included demographic and lifestyle factors: sex, age, body mass index (BMI), and smoking status; comorbidities: hypertension, diabetes mellitus (DM), previous stroke or transient ischemic attack (TIA), coronary heart disease (CHD), hyperlipidemia, and atrial fibrillation (AF); clinical assessment indicators: stroke etiology, mRS scores at admission and discharge, and National Institutes of Health Stroke Scale (NIHSS) score at admission; and laboratory parameters: white blood cell count (WBC), hemoglobin (HGB), hematocrit (HCT), fibrinogen (FIB), platelet count (PLT), mean corpuscular volume (MCV), triglycerides (TG), total cholesterol (TC), high‐density lipoprotein cholesterol (HDL‐C), low‐density lipoprotein cholesterol (LDL‐C), blood urea nitrogen (BUN), serum creatinine (Scr), alanine aminotransferase (ALT), aspartate aminotransferase (AST), fasting blood glucose (FBG), and hs‐CRP. Prediabetes mellitus (Pre‐DM) was defined as a FBG level of 100–125 mg/dL or an HbA1c level of 5.7%–6.4%. Normal glucose regulation (NGR) was defined as FBG < 100 mg/dL and HbA1c < 5.7% (ElSayed et al. 2023).

### 2.5 Statistical Analysis

The extent of missing data is detailed in Table S1. To address missing values and reduce bias, we excluded incomplete data. Baseline characteristics are presented as the means ± standard deviations (SDs) or medians with interquartile ranges (IQRs) for continuous variables and as frequencies with percentages for categorical variables. ANOVA was used for normally distributed quantitative variables, the Kruskal‒Wallis test was used for nonnormally distributed variables, and the chi‐square test was used for categorical variables.

The participants were divided into three groups based on the tertiles of the CTI: quartile (Q)1 < 3.6; Q2, 3.6–4.2; and Q3 > 4.2. The CTI was also assessed as a continuous variable to increase the robustness of our results. To investigate potential collinearity between the CTI and other covariates, we examined the generalized variance inflation factor (GVIF) and adjusted GVIF (Table S2). The analysis indicated no presence of collinearity. Logistic regression models were used to investigate the relationships between the CTI and adverse outcomes. Four distinct models were developed for a comprehensive analysis: a crude model without adjustments; Model 1, adjusted for age and sex; Model 2, additionally adjusted for BMI, WBC, HGB, AST, ALT, BUN, and LDL; and Model 3, further adjusted for smoking status, previous stroke/TIA, hypertension, DM, hyperlipidemia, AF, CHD, stroke etiology, and NIHSS score at admission. Furthermore, a fully adjusted restricted cubic spline (RCS) analysis was conducted to investigate the dose‒response relationship between the CTI and adverse outcomes. Additionally, we evaluated the relationship between the CTI and the risk of unfavorable outcomes across different sexes and glucose metabolism statuses, categorized as NGR, Pre‐DM, and DM. Subgroup analyses were performed to assess whether the effect of the CTI on unfavorable outcomes in AIS patients varied across diverse populations. These analyses were stratified by several factors, including sex, age, BMI, smoking status, hypertension, hyperlipidemia, DM, CHD, AF, and previous stroke/TIA.

To further validate the robustness of our findings, we conducted sensitivity analyses. First, we excluded participants with hypertension, hyperlipidemia, or CHD. Second, we categorized the CTI into two groups on the basis of the median and employed several propensity score methods, including propensity score adjustment, propensity score matching (nearest neighbor matching with a caliper width of 0.2 SDs), inverse probability of treatment weighting (IPTW), standardized mortality ratio weighting (SMRW), a pairwise algorithm (PA), and overlap weighting (OW). Propensity scores were estimated via logistic regression, adjusting for all relevant covariates. Finally, we calculated *E* values to quantify the minimum strength of association required between unmeasured confounders, the CTI, and adverse outcomes to explain the observed associations. The *E* value was calculated as *E* = RR + sqrt[RR × (RR−1)] (VanderWeele and Ding 2017). All the statistical analyses were performed via R software version 4.2.2, with statistical significance set at *p* < 0.05.

## 3 Results

### 3.1 Baseline Characteristics

Of the 1906 patients, 1485 (77.8%) were included (Table S3). Baseline characteristics were comparable between included and excluded patients, with no significant differences observed (all *p* > 0.05). Table 1 presents baseline data for the 1485 patients with AIS, stratified by tertiles of the CTI. Patients in the highest CTI tertile (Q3) presented a significantly greater BMI and prevalence rates of smoking, DM, and AF (all *p* < 0.001). There were also significant differences in baseline stroke severity across CTI tertiles, with patients in Q3 being more likely to present with higher NIHSS scores and worse mRS scores at admission (*p* < 0.001). The proportion of NGR decreased across tertiles, whereas the prevalence of DM increased (*p* < 0.001). Laboratory parameters associated with inflammation and metabolic dysfunction, including WBC, hs‐CRP, FBG, and TG, were significantly elevated at Q3 (*p* < 0.001). Conversely, the HDL‐C level was significantly lower in the highest CTI tertile (*p* < 0.001). No significant differences were observed in age distribution, prior stroke or TIA history, CHD, or TC across CTI tertiles. A comparison of baseline characteristics between patients with favorable and unfavorable outcomes is presented in Table S4.

**TABLE 1:** Patient demographics and baseline characteristics.

Characteristic	CTI quartiles				*p*
	Total	Q1	Q2	Q3	
Participants	1485	495	495	495	
Sex					0.116
Male	913 (61.5)	286 (57.8)	313 (63.2)	314 (63.4)	
Female	572 (38.5)	209 (42.2)	182 (36.8)	181 (36.6)	
Age (years)					0.113
< 60	324 (21.8)	103 (20.8)	102 (20.6)	119 (24)	
60 to < 70	393 (26.5)	138 (27.9)	138 (27.9)	117 (23.6)	
70 to < 80	529 (35.6)	178 (36)	187 (37.8)	164 (33.1)	
≥ 80	239 (16.1)	76 (15.4)	68 (13.7)	95 (19.2)	
BMI (kg/m2)	23.5 ± 3.3	22.9 ± 3.0	23.6 ± 3.2	23.9 ± 3.5	< 0.001
Smoking, n (%)					< 0.001
No	886 (59.7)	329 (66.5)	281 (56.8)	276 (55.8)	
Yes	599 (40.3)	166 (33.5)	214 (43.2)	219 (44.2)	
Hypertension, *n* (%)					0.059
No	540 (36.4)	198 (40)	180 (36.4)	162 (32.7)	
Yes	945 (63.6)	297 (60)	315 (63.6)	333 (67.3)	
NGR, *n* (%)					< 0.001
No	1099 (74.0)	296 (59.8)	384 (77.6)	419 (84.6)	
Yes	386 (26.0)	199 (40.2)	111 (22.4)	76 (15.4)	
Pre‐DM, *n* (%)					< 0.001
No	706 (47.5)	260 (52.5)	202 (40.8)	244 (49.3)	
Yes	779 (52.5)	235 (47.5)	293 (59.2)	251 (50.7)	
DM, *n* (%)					< 0.001
No	1029 (69.3)	389 (78.6)	359 (72.5)	281 (56.8)	
Yes	456 (30.7)	106 (21.4)	136 (27.5)	214 (43.2)	
Previous stroke/TlA, *n* (%)					0.972
No	1177 (79.3)	391 (79)	394 (79.6)	392 (79.2)	
Yes	308 (20.7)	104 (21)	101 (20.4)	103 (20.8)	
CHD, *n* (%)					0.202
No	1310 (88.2)	445 (89.9)	438 (88.5)	427 (86.3)	
Yes	175 (11.8)	50 (10.1)	57 (11.5)	68 (13.7)	
Hyperlipidemia, *n* (%)				0.061	3.498
No	934 (62.9)	658 (61.4)	276 (66.7)		
Yes	551 (37.1)	413 (38.6)	138 (33.3)		
Atrial fibrillation, *n* (%)				< 0.001	30.215
No	1172 (78.9)	884 (82.5)	288 (69.6)		
Yes	313 (21.1)	187 (17.5)	126 (30.4)		
Stroke etiology, *n* (%)					0.077
LAA	490 (33.0)	168 (33.9)	155 (31.4)	167 (33.7)	
SVO	283 (19.1)	108 (21.8)	96 (19.4)	79 (16)	
CE	374 (25.2)	114 (23)	132 (26.7)	128 (25.9)	
Other determined	124 (8.4)	32 (6.5)	37 (7.5)	55 (11.1)	
Undetermined	213 (14.4)	73 (14.7)	74 (15)	66 (13.3)	
mRS at admission, *n* (%)					< 0.001
0	1092 (73.6)	391 (79)	361 (72.9)	340 (68.8)	
1	136 (9.2)	42 (8.5)	49 (9.9)	45 (9.1)	
2	82 (5.5)	27 (5.5)	27 (5.5)	28 (5.7)	
3	77 (5.2)	22 (4.4)	28 (5.7)	27 (5.5)	
4	53 (3.6)	9 (1.8)	18 (3.6)	26 (5.3)	
5	44 (3.0)	4 (0.8)	12 (2.4)	28 (5.7)	
mRS at admission, *n* (%)					< 0.001
≤ 2	1071 (72.1)	395 (79.8)	372 (75.2)	304 (61.4)	
≥ 3	414 (27.9)	100 (20.2)	123 (24.8)	191 (38.6)	
NIHSS score at admission, *n* (%)					< 0.001
≤ 5	919 (61.9)	344 (69.5)	324 (65.5)	251 (50.7)	
5 to ≤ 13	426 (28.7)	128 (25.9)	131 (26.5)	167 (33.7)	
> 13	140 (9.4)	23 (4.6)	40 (8.1)	77 (15.6)	
Laboratory parameters					
WBC (10^9^/L)	8.2 ± 3.0	7.5 ± 2.3	8.0 ± 2.7	9.1 ± 3.5	< 0.001
HGB (g/dL)	13.5 ± 2.0	13.5 ± 1.8	13.8 ± 1.9	13.2 ± 2.2	< 0.001
HCT (%)	40.2 ± 5.5	40.3 ± 4.9	41.0 ± 5.2	39.2 ± 6.2	< 0.001
FIB (mg/L)	332.4 ± 90.1	297.2 ± 54.7	321.8 ± 64.6	378.3 ± 117.4	< 0.001
PLT, mean ± SD	225.2 ± 69.8	222.4 ± 66.0	223.2 ± 62.8	230.0 ± 79.3	0.164
MCV, mean ± SD	92.9 ± 5.3	93.0 ± 5.4	93.3 ± 5.2	92.4 ± 5.2	0.02
TG (mg/dL)	109.8 ± 54.7	82.4 ± 30.6	107.2 ± 39.4	139.8 ± 69.5	< 0.001
TC, mean ± SD	180.5 ± 44.2	178.6 ± 38.9	182.8 ± 41.7	180.3 ± 51.0	0.327
HDL‐C (mg/dL)	46.6 ± 13.6	51.7 ± 14.0	47.0 ± 11.8	41.2 ± 12.9	< 0.001
LDL‐C (mg/dL)	108.8 ± 38.4	105.6 ± 33.0	111.2 ± 37.9	109.7 ± 43.4	0.06
BUN (mg/dL)	17.5 ± 8.9	16.4 ± 7.4	17.0 ± 7.6	19.0 ± 11.0	< 0.001
Scr (mg/dL)	0.9 (0.7, 1.1)	0.9 (0.7, 1.0)	0.9 (0.7, 1.1)	0.9 (0.7, 1.2)	0.001
ALT (U/L)	18.0 (13.0, 26.0)	17.0 (13.0, 24.0)	18.0 (14.0, 26.0)	19.0 (13.0, 29.0)	0.035
AST (U/L)	18.0 (13.0, 26.0)	17.0 (13.0, 24.0)	18.0 (14.0, 26.0)	19.0 (13.0, 29.0)	0.035
FBG (mg/dL)	106.6 ± 38.6	90.1 ± 24.0	104.6 ± 29.3	125.1 ± 49.2	< 0.001
hs‐CRP (mg/L)	0.2 (0.1, 0.5)	0.0 (0.0, 0.1)	0.2 (0.1, 0.3)	0.9 (0.3, 3.3)	< 0.001
CTI	4.0 ± 0.8	3.1 ± 0.3	3.9 ± 0.2	4.8 ± 0.5	< 0.001

*Note*: Variables are presented as mean ± SD, median (IQR), or *n* (%).

Abbreviations: ALT, alanine aminotransferase; AST, aspartate aminotransferase; BMI, body mass index; BUN, blood urea nitrogen; CHD, coronary heart disease; CTI, C‐reactive protein‐triglyceride glucose index; DM, diabetes mellitus; FBG, fasting blood glucose; FIB, fibrinogen; HCT, hematocrit; HDL‐C, high‐density lipoprotein cholesterol; HGB, hemoglobin; hs‐CRP, high‐sensitivity C‐reactive protein; LDL‐C, low‐density lipoprotein cholesterol; MCV, mean corpuscular volume; mRS, modified Rankin scale; NGR, normal glucose regulation; NIHSS, National Institutes of Health Stroke Scale; PLT, platelet; Pre‐DM, prediabetes mellitus; Scr, serum creatinine; TC, total cholesterol; TG, triglyceride; TIA, transient ischemic attack; WBC, white blood cell.

### 3.2 Associations Between the CTI and Unfavorable Outcomes in Patients With AIS

**FIGURE 2:**
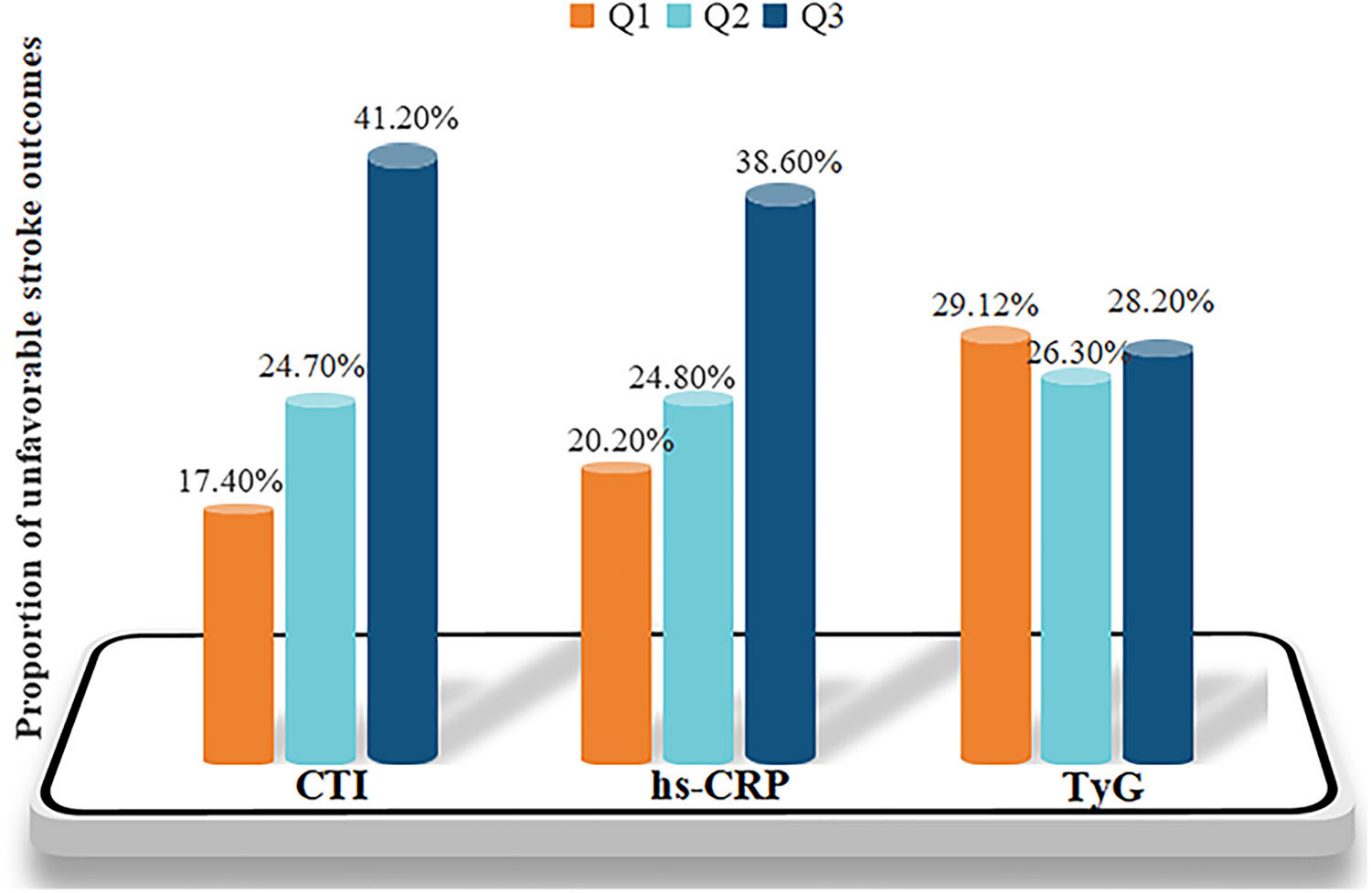
Proportion of unfavorable outcomes in AIS by CTI, hs‐CRP, and TyG quartiles.

**FIGURE 3:**
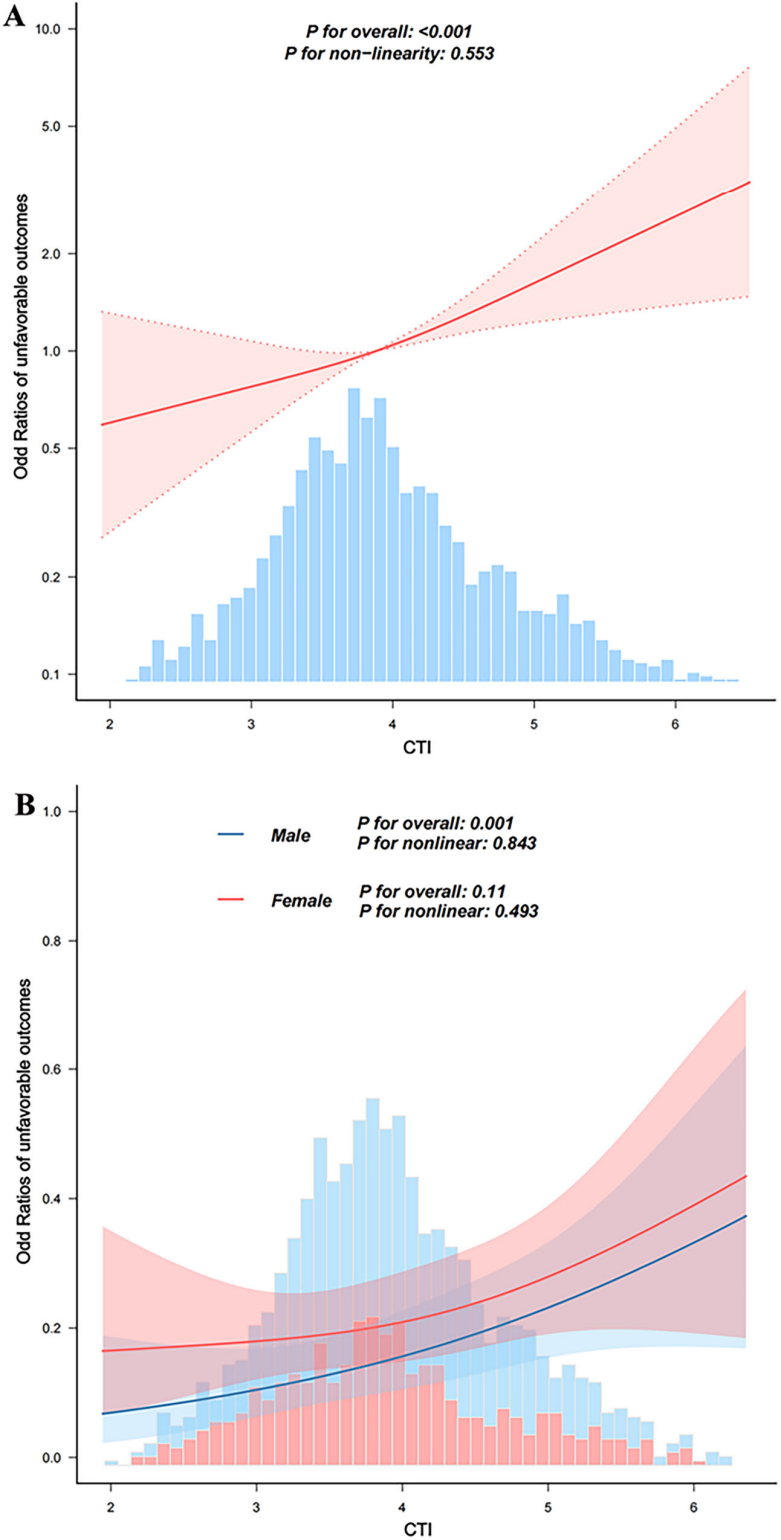
Association of the CTI index and the unfavorable outcomes in AIS according to gender. (A) Total participants; (B) male and female. Adjusted for age, sex, BMI, WBC, HGB, AST, ALT, BUN, LDL, smoking, previous stroke/TIA, hypertension, DM, hyperlipidemia, AF, CHD, stroke etiology, and NIHSS score at admission.

The incidence of unfavorable outcomes in AIS patients increased progressively across tertiles for CTI, with 85 (17.4%) in Q1, 122 (24.7%) in Q2, and 207 (41.2%) in Q3 (Figure 2). A similar pattern was observed for hs‐CRP, with 100 (20.2%) in Q1, 123 (24.8%) in Q2, and 191 (38.6%) in Q3, whereas the distribution for TyG remained relatively stable (Q1: 29.1%, Q2: 26.3%, and Q3: 28.2%). Each 1‐unit increase in CTI was significantly associated with a greater risk of poor outcomes in both unadjusted (odds ratio [OR] = 1.974, 95% CI: 1.694–2.299, *p *< 0.001) and fully adjusted models (Model 3: OR = 1.484, 95% CI: 1.229–1.793, *p*<0.001), accounting for demographic characteristics, laboratory parameters, and clinical comorbidities (Table 2). Quartile‐based analysis further revealed a significantly greater risk in Q3 than in Q1 (OR = 1.981, 95% CI: 1.385–2.834, *p *< 0.001; *p* for trend = 0.0074). The fitted curve (Figure 3A) further confirmed the positive association between the CTI and poor outcomes in patients with AIS (*p* = 0.001). In contrast, hs‐CRP showed a consistent but weaker association (Model 3: OR = 1.06, 95% CI: 1.012–1.111, *p* = 0.0136), and TyG did not reach statistical significance in the fully adjusted model (OR = 1.086, 95% CI: 0.826–1.429, *p* = 0.5551) (Table S5). These results suggest that the CTI, as a composite index integrating inflammatory and metabolic status, may serve as a more robust prognostic marker for unfavorable outcome risk stratification in AIS patients than the CRP and TyG indices do.

**TABLE 2:** Association between CTI and unfavorable outcomes 3 months after stroke in different models.

Characteristic	Event (*n*%)	Crude model		Model 1		Model 2		Model 3	
		OR (95%CI)	*p*	OR (95%CI)	*p*	OR (95%CI)	*p*	OR (95%CI)	*p*
CTI (per 1 unit)	414 (27.9%)	1.974 (1.694–2.299)	< 0.001	1.97 (1.69–2.3)	< 0.001	1.606 (1.392–1.852)	<0.001	1.484 (1.229–1.793)	< 0.001
CTI									
Q1 (< 3.6)	93 (18.8%)	1 (Ref)		1 (Ref)		1 (Ref)		1 (Ref)	
Q2 (3.6–4.2)	121 (24.4%)	1.398 (1.031–1.897)	0.031	1.4 (1.03–1.92)	0.034	1.421 (1.039–1.945)	0.028	1.217 (0.848–1.748)	0.287
Q3 (> 4.2)	200 (40.4%)	2.931 (2.197–3.91)	< 0.001	2.99 (2.22–4.02)	< 0.001	2.481 (1.81–3.401)	< 0.001	1.981 (1.385–2.834)	< 0.001
*p* for trend		1.74 (1.505–2.012)	< 0.001	1.75 (1.51–2.04)	< 0.001	1.582 (1.351–1.854)	< 0.001	1.418 (1.184–1.697)	< 0.001

*Note*: Crude model: we did not adjust for other covariates; Model 1: age and sex; Model 2: age, sex, BMI, WBC, HGB, AST, ALT, BUN, and LDL; Model 3: age, sex, BMI, WBC, HGB, AST, ALT, BUN, LDL, smoking, previous stroke/TIA, hypertension, DM, hyperlipidemia, AF, CHD, stroke etiology, and NIHSS score at admission.

**FIGURE 4:**
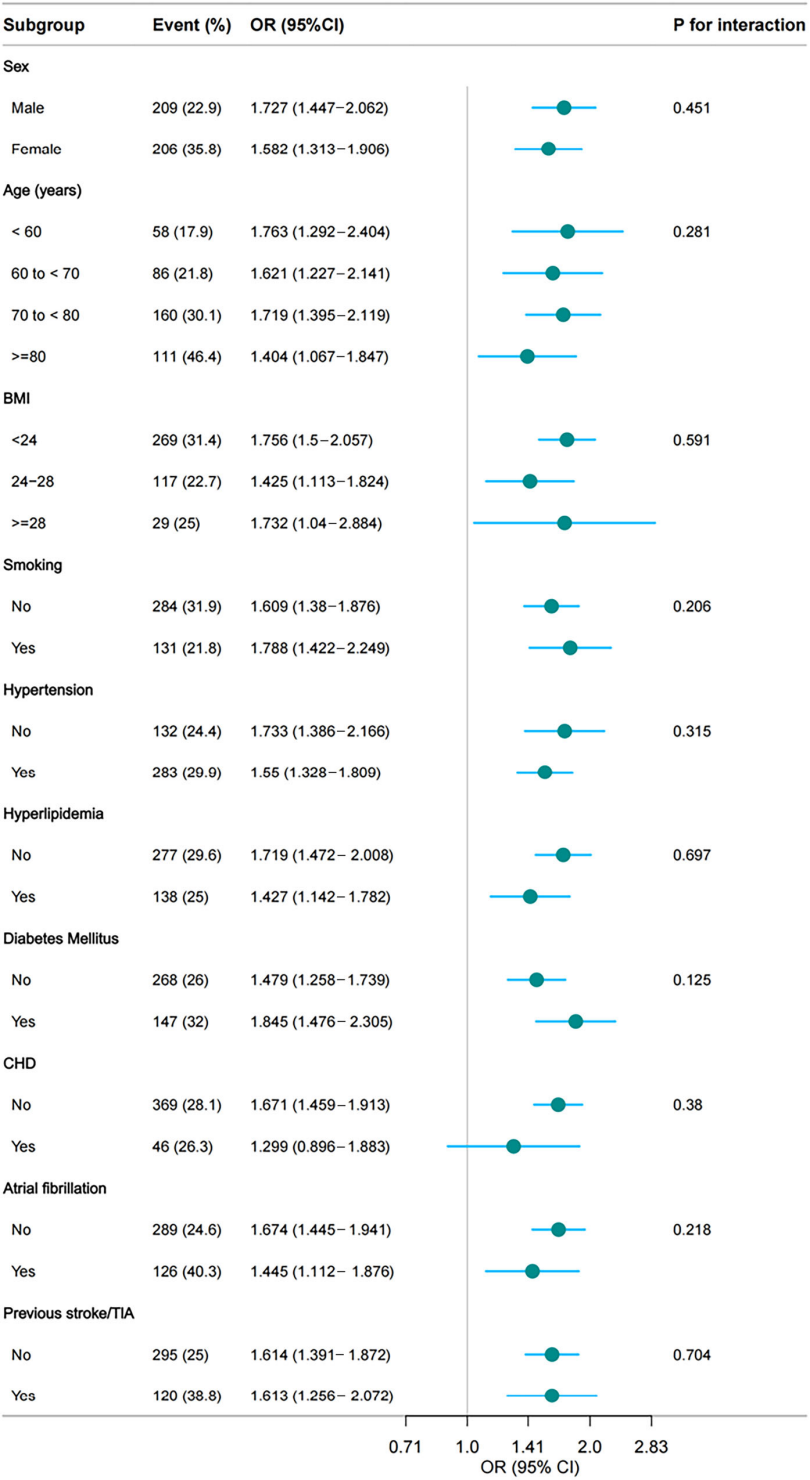
Subgroup and interaction analyses of the association between CTI and unfavorable outcomes of AIS. Adjusted for age, sex, BMI, WBC, HGB, AST, ALT, BUN, LDL, smoking, previous stroke/TIA, hypertension, DM, hyperlipidemia, AF, CHD, stroke etiology, and NIHSS score at admission.

The association between the CTI and unfavorable outcomes 3 months after stroke was significant in males and marginally significant in females after adjusting for confounders (Table S6). In males, the OR decreased from 2.149 (95% CI 1.736–2.659, *p *< 0.001) in the crude model to 1.591 (95% CI 1.235–2.050, *p* = 0.0003) in Model 3. In females, the OR decreased from 1.892 (95% CI 1.513–2.366, *p *< 0.001) in the crude model to 1.347 (95% CI 1.004–1.807, *p* = 0.047) in Model 3. Interaction analysis revealed no significant difference between males and females (*p* for interaction = 0.451) (Figure 4). The RCS analysis revealed a significant linear relationship in males (*p* for overall = 0.001) but not in females (*p* for overall = 0.11) (Figure 3B).

### 3.3 Associations Between the CTI and Unfavorable Outcomes in AIS Patients According to Glycemic Status

**FIGURE 5:**
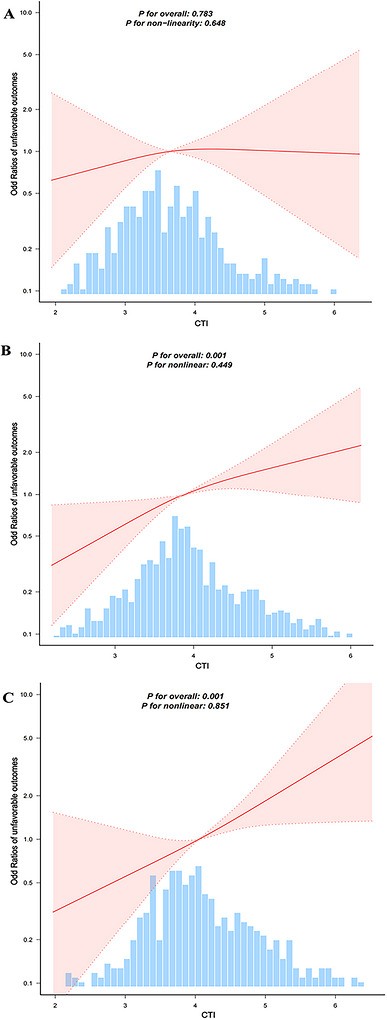
Association of the CTI index and the unfavorable outcomes of AIS according to glucose metabolic states. (A) Participants with NGR. (B) Participants with Pre‐DM. (C) Participants with DM. Adjusted for age, sex, BMI, WBC, HGB, AST, ALT, BUN, LDL, smoking, previous stroke/TIA, hypertension, DM, hyperlipidemia, AF, CHD, stroke etiology, and NIHSS score at admission.

At 3 months after AIS, 86 (22.3%) participants with NGR, 273 (29%) participants with Pre‐DM, and 147 (32%) participants with DM experienced unfavorable outcomes (Figure S1). The proportion of unfavorable stroke outcomes varied across the CTI categories in the NGR, Pre‐DM, and DM groups. As shown in Table 3, a significant association between the CTI and stroke risk was observed in individuals with Pre‐DM and DM in Model 3. However, no significant difference was found in individuals with NGR (all *p* > 0.05). Specifically, for each 1‐unit increase in CTI, the risk of unfavorable outcomes increased by 63.4% in individuals with Pre‐DM (OR: 1.634, 95% CI 1.267–2.108) and by 81.9% in individuals with DM (OR: 1.819, 95% CI 1.315–2.517). The RCS analysis revealed a significant linear relationship between the CTI and unfavorable outcomes in individuals with Pre‐DM (Figure 5B) and in individuals with DM (Figure 5C). However, no significant dose‒response correlation between the CTI and unfavorable outcomes was observed in individuals with NGR (Figure 5A).

**TABLE 3:** Associations between the CTI and unfavorable outcomes after stroke across glucose regulation states.

GMS	Characteristic	Event (*n*%)	Crude model		Model 1		Model 2		Model 3	
			OR	*p*	OR (95%CI)	*p*	OR	*p*	OR	*p*
			(95%CI)				(95%CI)		(95%CI)	
NGR	CTI (per 1 unit)	86 (22.3%)	1.522 (1.115–2.078)	0.008	1.47 (1.07–2.03)	0.018	1.451 (1.01–2.084)	0.044	1.12 (0.772–1.625)	0.551
	CTI									
	Q1	24 (18.6%)	1 (Ref)		1 (Ref)		1 (Ref)		1 (Ref)	
	Q2	25 (19.5%)	1.062 (0.57–1.979)	0.85	1.05 (0.55–2)	0.889	0.97 (0.506–1.86)	0.927	1.097 (0.531–2.265)	0.802
	Q3	37 (28.7%)	1.76 (0.98–3.158)	0.058	1.75 (0.95–3.21)	0.073	1.622 (0.849–3.098)	0.143	1.321 (0.645–2.704)	0.446
	*p* for trend		1.341 (0.996–1.806)	0.053	1.34 (0.98–1.82)	0.065	1.283 (0.923–1.783)	0.139	1.151 (0.804–1.648)	0.442
Pre‐DM	CTI (per 1 unit)	273 (29%)	1.987 (1.622–2.434)	< 0.001	2.01 (1.63–2.48)	< 0.001	1.776 (1.422–2.219)	< 0.001	1.634 (1.267–2.108)	< 0.001
	CTI									
	Q1	58 (18.5%)	1 (Ref)		1 (Ref)		1 (Ref)		1 (Ref)	
	Q2	89 (28.5%)	1.755 (1.204–2.557)	0.003	1.74 (1.19–2.57)	0.005	1.821 (1.23–2.697)	0.002	1.691 (1.066–2.683)	0.025
	Q3	125 (39.9%)	2.923 (2.031–4.207)	< 0.001	3.04 (2.08–4.43)	< 0.001	2.588 (1.747–3.834)	< 0.001	2.422 (1.529–3.837)	< 0.001
	*p* for trend		1.706 (1.425–2.043)	< 0.001	1.74 (1.45–2.1)	< 0.001	1.599 (1.317–1.941)	< 0.001	1.549 (1.234–1.946)	< 0.001
DM	CTI (per 1 unit)	147 (32%)	2.376 (1.805–3.129)	< 0.001	2.36 (1.79–3.12)	< 0.001	2.111 (1.54–2.892)	< 0.001	1.819 (1.315–2.517)	< 0.001
	CTI									
	Q1	29 (19.1%)	1 (Ref)		1 (Ref)		1 (Ref)		1 (Ref)	
	Q2	44 (28.9%)	1.728 (1.012–2.951)	0.045	1.78 (1.04–3.06)	0.035	1.956 (1.119–3.422)	0.019	1.592 (0.851–2.976)	0.146
	Q3	74 (48.7%)	4.024 (2.405–6.732)	< 0.001	4.07 (2.42–6.85)	< 0.001	3.32 (1.87–5.894)	< 0.001	2.71 (1.445–5.083)	0.002
	*p* for trend		2.032 (1.571–2.63)	< 0.001	2.04 (1.57–2.64)	< 0.001	1.817 (1.366–2.417)	< 0.001	1.649 (1.205–2.257)	0.002

*Note*: Crude model: we did not adjust for other covariates; Model 1: age and sex; Model 2: age, sex, BMI, WBC, HGB, AST, ALT, BUN, and LDL; Model 3: age, sex, BMI, WBC, HGB, AST, ALT, BUN, LDL, smoking, previous stroke/TIA, hypertension, hyperlipidemia, AF, CHD, stroke etiology, and NIHSS score at admission.

As shown in Table 4, in male participants, a significant association was found between the CTI and unfavorable outcomes in individuals with Pre‐DM (OR 2.7, 95% CI 1.97–3.69) and DM (OR 1.571, 95% CI 1.011–2.443), whereas no significant difference was observed in those with NGR. In female participants, no significant associations were found in individuals with NGR or Pre‐DM (all *p* > 0.05), whereas a significant association was observed in individuals with DM (OR 3.001, 95% CI 1.672–5.384).

**TABLE 4:** Sex‐stratified associations between the CTI and unfavorable outcomes after stroke across glucose regulation states.

Sex	GMS	Characteristic	Event(*n*%)	Crude model		Model 1		Model 2		Model 3	
				OR (95%CI)	*p*	OR (95%CI)	*p*	OR (95%CI)	*p*	OR (95%CI)	*p*
Male	NGR	CTI (per 1 unit)	47 (18.8%)	1.503 (0.987–2.288)	0.057	1.51 (0.99–2.31)	0.054	1.719 (1.06–2.785)	0.028	1.135 (0.695–1.853)	0.614
		CTI									
		Q1	12 (14.5%)	1 (Ref)		1 (Ref)		1 (Ref)		1 (Ref)	
		Q2	14 (16.9%)	1.2 (0.519–2.779)	0.670	1.3 (0.55–3.04)	0.551	1.306 (0.545–3.128)	0.549	1.319 (0.495–3.52)	0.580
		Q3	21 (25%)	1.972 (0.898–4.329)	0.090	2.19 (0.98–4.91)	0.056	2.498 (1.052–5.935)	0.038	1.616 (0.615–4.25)	0.330
		*p* for trend		1.42 (0.955–2.112)	0.083	1.49 (0.99–2.24)	0.053	1.597 (1.031–2.474)	0.036	1.269 (0.785–2.052)	0.331
	Pre‐DM	CTI (per 1 unit)	131 (23.4%)	2.699 (1.983–3.675)	< 0.001	2.2 (1.68–2.88)	< 0.001	2.507 (1.776–3.54)	<0.001	2.7 (1.97–3.69)	< 0.001
		CTI									
		Q1	20 (10.7%)	1 (Ref)		1 (Ref)		1 (Ref)		1 (Ref)	
		Q2	42 (22.5%)	2.419 (1.358–4.307)	0.003	2.28 (1.27–4.09)	0.006	2.178 (1.196–3.965)	0.011	2.2 (1.079–4.483)	0.030
		Q3	69 (36.9%)	4.883 (2.815–8.469)	< 0.001	4.79 (2.75–8.34)	< 0.001	4.17 (2.336–7.443)	< 0.001	4.84 (2.474–9.471)	< 0.001
		*p* for trend		2.178 (1.675–2.834)	< 0.001	2.17 (1.67–2.84)	< 0.001	2.025 (1.529–2.68)	< 0.001	2.2 (1.589–3.047)	< 0.001
	DM	CTI (per 1 unit)	76 (27.5%)	2.175 (1.52–3.113)	< 0.001	2.15 (1.5–3.09)	< 0.001	1.958 (1.266–3.029)	0.003	1.571 (1.011–2.443)	0.045
		CTI									
		Q1	14 (15.2%)	1 (Ref)		1 (Ref)		1 (Ref)		1 (Ref)	
		Q2	25 (27.5%)	2.11 (1.015–4.387)	0.0455	2.15 (1.03–4.51)	0.042	2.139 (0.982–4.66)	0.0555	1.943 (0.797–4.738)	0.144
		Q3	37 (40.2%)	3.748 (1.852–7.587)	2e‐04	3.81 (1.87–7.76)	< 0.001	3.021 (1.344–6.792)	0.0075	2.41 (0.987–5.887)	0.0535
		*p* for trend		1.918 (1.36–2.705)	2e‐04	1.93 (1.37–2.73)	< 0.001	1.719 (1.156–2.557)	0.0075	1.525 (0.986–2.358)	0.0579
Female	NGR	CTI (per 1 unit)	39 (28.7%)	1.658 (1.033–2.662)	0.0362	1.53 (0.91–2.57)	0.106	1.282 (0.703–2.338)	0.4178	0.927 (0.462–1.859)	0.8307
		CTI									
		Q1	10 (22.2%)	1 (Ref)		1 (Ref)		1 (Ref)		1 (Ref)	
		Q2	13 (28.9%)	2.452 (0.881–6.825)	0.086	1.9 (0.6–5.97)	0.274	1.601 (0.491–5.224)	0.4351	3.11 (0.792–12.212)	0.1039
		Q3	16 (34.8%)	3.49 (1.283–9.489)	0.0143	3.11 (1.06–9.19)	0.04	2.424 (0.743–7.911)	0.1422	2.365 (0.585–9.561)	0.227
		*p* for trend		1.816 (1.125–2.932)	0.0147	1.75 (1.03–2.96)	0.037	1.553 (0.865–2.789)	0.1401	1.454 (0.748–2.826)	0.2694
	Pre‐DM	CTI (per 1 unit)	142 (37.5%)	1.615 (1.226–2.127)	7e‐04	1.52 (1.14–2.02)	0.004	1.511 (1.091–2.093)	0.0129	1.138 (0.766–1.691)	0.5212
		CTI									
		Q1	37 (29.4%)	1 (Ref)		1 (Ref)		1 (Ref)		1 (Ref)	
		Q2	49 (39.2%)	1.551 (0.917–2.622)	0.1016	1.5 (0.87–2.58)	0.143	1.777 (1.009–3.131)	0.0464	1.801 (0.882–3.676)	0.1061
		Q3	55 (43.7%)	1.863 (1.107–3.136)	0.0191	1.62 (0.94–2.79)	0.081	1.655 (0.904–3.028)	0.1023	0.871 (0.403–1.882)	0.7249
		*p* for trend		1.36 (1.05–1.761)	0.0196	1.27 (0.97–1.66)	0.083	1.293 (0.96–1.743)	0.0906	0.953 (0.656–1.385)	0.8023
	DM	CTI (per 1 unit)	71 (38.8%)	2.865 (1.83–4.485)	< 0.001	3.03 (1.9–4.83)	< 0.001	2.877 (1.711–4.837)	1e‐04	3.001 (1.672–5.384)	2e‐04
		CTI									
		Q1	13 (21.7%)	1 (Ref)		1 (Ref)		1 (Ref)		1 (Ref)	
		Q2	21 (35%)	1.947 (0.865–4.383)	0.1077	1.88 (0.83–4.27)	0.129	2.03 (0.869–4.741)	0.102	1.925 (0.701–5.283)	0.2035
		Q3	37 (60.7%)	5.574 (2.503–12.413)	< 0.001	5.9 (2.59–13.46)	< 0.001	5.322 (2.141–13.226)	3e‐04	6.469 (2.198–19.037)	7e‐04
		*p* for trend		2.393 (1.601–3.578)	< 0.001	2.46 (1.62–3.73)	< 0.001	2.309 (1.462–3.647)	3e‐04	2.535 (1.475–4.357)	8e‐04

*Note*: Crude model: we did not adjust for other covariates; Model 1: age; Model 2: age, BMI, WBC, HGB, AST, ALT, BUN, and LDL; Model 3: age, BMI, WBC, HGB, AST, ALT, BUN, LDL, smoking, previous stroke/TIA, hypertension, hyperlipidemia, AF, CHD, stroke etiology, and NIHSS score at admission.

### 3.4 Subgroup Analysis

Subgroup analysis revealed that for each 1‐unit increase in the CTI, the OR for unfavorable outcomes in AIS patients remained stable across all subgroups, with no statistically significant interaction effects (*p* > 0.05) (Figure 4). Sex, age, BMI, smoking, hypertension, hyperlipidemia, DM, CHD, AF, and previous stroke/TlA did not significantly modify this association.

### 3.5 Sensitivity Analysis

To assess the robustness of the findings, multiple sensitivity analyses were conducted. First, the results remained consistent even after excluding hypertension, hyperlipidemia, and CHD (Table S7). Second, propensity score matching and various weighting methods (IPTW, SMRW, PA, and OW) yielded consistent results, with ORs ranging from 1.473 to 1.517, and all *p* values remained significant (*p* < 0.05), further supporting the robustness of the findings. (Figure S2). Additionally, the E value for the CTI was calculated via Model 3, revealing an *E* value of 2.67 (Figure S3). This *E*‐value represents the minimum strength of association that an unmeasured confounder would need to fully account for the observed hazard ratios. It should be interpreted with caution, as it does not rule out the presence of residual confounding.

## 4 Discussion

This study is the first to investigate the relationship between the CTI and unfavorable prognosis in patients with AIS, considering both sex and glucose metabolic status. We found a significant positive correlation between the CTI and adverse outcomes, particularly in males with Pre‐DM and DM. However, no such association was observed in individuals with NGR. Furthermore, the CTI demonstrated superior predictive performance compared with traditional markers such as hs‐CRP and TyG, making it a more reliable tool for AIS prognosis risk stratification. These findings provide preliminary evidence for the potential of the CTI as a prognostic tool for AIS.

The CTI is composed of two key indicators: the hs‐CRP level and the TyG index. A meta‐analysis revealed that hs‐CRP is closely associated with mortality, stroke recurrence risk, and poor outcomes in stroke patients (Chen et al. 2023). Additionally, some studies have noted that hs‐CRP is associated with poor prognosis in AIS patients with large vessel occlusion and those who are undergoing mechanical thrombectomy (Kim et al. 2020). Moreover, TyG, a surrogate marker for insulin resistance, has been widely recognized (Vasques et al. 2011; Minh et al. 2021). Compared with traditional measurement methods such as the hyperinsulinemic‐euglycemic clamp (Wallace and Matthews 2002) and the homeostasis model assessment for insulin resistance (Jiang et al. 2025), TyG offers a simpler and more cost‐effective alternative (Jeong and Lee 2021; Dikaiakou et al. 2020). Previous studies have shown that the TyG score is associated with poor functional outcomes at discharge from the AIS (Miao et al. 2023); meta‐analyses further confirmed the significant correlation between the TyG score and poor functional outcomes in AIS patients (Ma et al. 2022). Furthermore, the TyG index has been found to predict 1‐year recurrent stroke risk in female AIS patients (Guo et al. 2024). Although TyG alone was not significantly associated with AIS outcomes in this study, its combination with hs‐CRP in the CTI notably enhanced the predictive performance. CTI yielded higher ORs for poor AIS outcomes than did CRP or TyG alone in both the continuous and categorical analyses. These findings underscore the value of combining inflammatory markers with indicators of insulin resistance in stroke prognostic models and highlight the clinical utility of the CTI. Notably, insulin resistance in the context of systemic inflammation may exert a particularly adverse influence on stroke recovery.

This study revealed a significant positive association between elevated CTI levels and poor prognosis in patients with AIS, with RCS analysis confirming a linear relationship. Although trends were examined across sex, the interaction between sex and CTI was not statistically significant. This indicates no clear difference between males and females. Stratified analyses based on glycemic status revealed that an elevated CTI was significantly associated with poor outcomes in patients with Pre‐DM and DM, with RCS analysis confirming a stable linear trend. However, no such association was observed in individuals with NGR, likely due to their relatively stable metabolic and inflammatory profiles and the absence of insulin resistance and systemic inflammation common in Pre‐DM and DM populations (ElSayed et al. 2023; Zhang et al. 2021; Herrerías‐García et al. 2025). These findings indicate that the prognostic value of the CTI is modulated by glycemic status, underscoring the importance of individualized interpretation and intervention strategies in AIS management on the basis of metabolic profiles.

We conducted a comprehensive analysis of the relationship between the CTI and poor outcomes in AIS patients across different sexes and glycemic statuses. The results showed that the CTI was significantly associated with adverse outcomes in male patients with pre‐DM and DM, suggesting that controlling CTI levels may have important clinical implications in this population. Among female patients with DM, the risk of poor prognosis was significantly greater in the highest CTI quartile than in the lowest quartile, indicating that elevated CTI levels also warrant attention in female patients with DM. Further subgroup analyses demonstrated that the associations between the CTI and poor outcomes remained consistent across age, sex, and comorbidity subgroups, supporting the robustness of our findings. To our knowledge, this is the first study to systematically examine the relationship between the CTI and AIS prognosis from the dual perspectives of sex and glycemic status, providing a novel potential biomarker for risk stratification in AIS patients.

The exact mechanisms underlying the association between the CTI and an unfavorable prognosis in AIS patients remain unclear, but several plausible explanations have been proposed. CTI may reflect a synergistic interaction between systemic inflammation and insulin resistance, two interrelated processes that can mutually reinforce each other, thereby amplifying inflammatory responses, compromising endothelial integrity, and increasing the risk of adverse outcomes after AIS (Amudi et al. 2021). Specifically, hs‐CRP, a well‐established marker of systemic inflammation, can interact with platelets, monocytes, and endothelial cells at sites of inflammation, activating the complement system and promoting leukocyte recruitment and platelet aggregation, which in turn contribute to microvascular dysfunction and exacerbate ischemic injury following AIS (Amudi et al. 2021; Yüksel et al. 2025; Perumalsamy et al. 2023; Owusu and Barrett 2021; McFadyen et al. 2020; Swastini et al. 2019; Prasad 2024). Concurrently, insulin resistance may impair nitric oxide bioavailability, leading to endothelial dysfunction, impaired vasodilation, and dysregulated coagulation, thus accelerating the progression of atherosclerosis (McFadyen et al. 2020; Swastini et al. 2019; Prasad 2024). Furthermore, elevated CTI levels are frequently observed in patients with metabolic comorbidities such as hypertension, diabetes, and obesity (Tang et al. 2024; Xu et al. 2024), reinforcing its potential role as an integrated marker of metabolic–inflammatory dysregulation. Overall, these mechanisms may account for the observed link between an elevated CTI and poor AIS prognosis, although further research is warranted to elucidate the underlying biological pathways involved.

This study is the first to identify a significant positive association between the CTI and adverse outcomes in AIS patients, underscoring its potential as a clinically valuable prognostic biomarker. To further evaluate the clinical relevance of the CTI, we examined this association across subgroups stratified by sex and glycemic status. Subgroup analyses across age, sex, and comorbidity status were performed to validate the stability of the association, thereby strengthening the robustness of the findings. Notably, the CTI is a clinically accessible indicator that has considerable potential for practical application and clinical translation.

However, several limitations of this study should be acknowledged. all components of the CTI (hs‐CRP, TG, and FBG) were measured only once at admission. In AIS, both inflammation and glucose metabolism are highly dynamic, and a single baseline measurement may not fully capture these temporal changes. Future studies incorporating serial measurements are warranted. Second, the study used the 3‐month mRS (mRS ≥ 3) as the sole outcome measure. The mRS is widely accepted for assessing poststroke functional status, but it captures only functional disability. It does not account for other clinically important outcomes, such as mortality, stroke recurrence, cognitive function, or quality of life. The CTI reflects both inflammation and insulin resistance, which may influence multiple aspects of recovery. Future studies incorporating a broader range of outcomes are warranted to fully assess its prognostic value. Third, data on reperfusion therapies, including intravenous thrombolysis and endovascular thrombectomy, were not available and could not be included in the analyses. These treatments are among the key determinants of functional outcomes after AIS, and their omission may have introduced confounding. Future studies should incorporate these therapeutic variables to more accurately assess the prognostic value of CTI. Fourth, The coefficient of ln(hs‐CRP) (0.412) in the CTI formula was originally derived from studies in cancer populations and has not been specifically validated in AIS patients. Therefore, the relative contribution of CRP in AIS may differ, which could affect the absolute predictive performance of the CTI. Future studies could reestimate the CRP weighting in the CTI based on AIS patient data using statistical modeling or machine learning approaches, to further improve the predictive accuracy and applicability of the CTI. Finally, the follow‐up period in this study was relatively short, limited to 3 months after AIS onset. The study population included only Korean patients from a single center, which may limit the generalizability of our findings. Therefore, further large‐scale, multicenter, and long‐term prospective studies are warranted to validate the prognostic value of CTI across diverse populations, clinical settings, and extended follow‐up periods.

## 5 Conclusions

This study demonstrated a significant association between elevated CTI levels and unfavorable prognosis at 3 months in patients with AIS. Further analyses revealed that this association was significant among males with Pre‐DM and DM, and a similar significant relationship was also observed in females with DM. However, no such association was found in individuals with NGR. These findings suggest that tailored management strategies based on sex and glycemic status may help reduce the risk of adverse outcomes in AIS patients.

## Author Contributions

**Huang Luwen**: methodology, software, formal analysis, data curation, writing – original draft. **Chen Qin**: revised the writing, methodology, investigation, data curation. **Xu Lei**: methodology, investigation, data curation. **Yu Ming**: conceptualization, methodology, writing – review and editing, supervision. **Li Linlin**: conceptualization, methodology, writing – review and editing, supervision, project.

## Funding

The authors have nothing to report.

## Ethics Statement

This study was approved by the institutional review board of Seoul National University Hospital (IRB NO. 1009‐062‐332), with a waiver of patient consent.

## Conflicts of Interest

The authors declare no conflicts of interest.

## Supporting information




**Supplementary Table S1**: brb371578‐sup‐0001‐TableS1.docx

**Supplementary Table S2**: brb371578‐sup‐0002‐TableS2.docx

**Supplementary Table S3**: brb371578‐sup‐0003‐TableS3.docx

**Supplementary Table S4**: brb371578‐sup‐0004‐TableS4.docx

**Supplementary Table S5**: brb371578‐sup‐0005‐TableS5.docx

**Supplementary Table S6**: brb371578‐sup‐0006‐TableS6.docx

**Supplementary Figure S1**: brb371578‐sup‐0007‐FigureS1.docx

**Supplementary Table S7**: brb371578‐sup‐0008‐TableS7.docx

**Supplementary Figure S2**: brb371578‐sup‐0009‐FigureS2.docx

**Supplementary Figure S3**: brb371578‐sup‐0010‐FigureS3.docx

## Data Availability

The data used in this study are publicly available and can be accessed through the PLoS ONE database at https://journals.plos.org/plosone/article?id https://doi.org/10.1371/journal.pone.0228738#sec019.
