# Supplementary material for: The Prognostic Role of C‐Reactive Protein–Triglyceride Glucose Index in Predicting Unfavorable Outcomes in Acute Ischemic Stroke: A Large‐Scale Cohort Study
Source: Brain Behav. 2026 Jul 9;16(7):e71578. doi: 10.1002/brb3.71578 (PMC13347318; doi:10.1002/brb3.71578)
Supplement: Supplementary file 1 — Supplementary Table S1: brb371578‐sup‐0001‐TableS1.docx [file BRB3-16-e71578-s002.docx]

| Table S1.  Distribution of variables with missing data. | | |
| --- | --- | --- |
| **Variables** | **Number of missi**ng | **Missing proporti**on |
| WBC | 1 | 0.07% |
| Stroke etiology | 1 | 0.07% |
| mRS at admission | 1 | 0.07% |

Note: WBC, white blood cell; mRS, modified rankin scale.
