# Supplementary material for: The Prognostic Role of C‐Reactive Protein–Triglyceride Glucose Index in Predicting Unfavorable Outcomes in Acute Ischemic Stroke: A Large‐Scale Cohort Study
Source: Brain Behav. 2026 Jul 9;16(7):e71578. doi: 10.1002/brb3.71578 (PMC13347318; doi:10.1002/brb3.71578)
Supplement: Supplementary file 2 — Supplementary Table S2: brb371578‐sup‐0002‐TableS2.docx [file BRB3-16-e71578-s010.docx]

| Table S2.  Assessment of collinearity among independent variables in the final regression model. | | | | |
| --- | --- | --- | --- | --- |
| **Variables** | **GVIF** | **Df** | **Adjusted GVIF** | **Collinearity** |
| Age | 1.307 | 1 | 1.143 | No |
| Sex | 1.338 | 3 | 1.05 | No |
| BMI | 1.255 | 1 | 1.12 | No |
| WBC | 1.496 | 1 | 1.223 | No |
| HGB | 1.161 | 1 | 1.078 | No |
| AST | 1.215 | 1 | 1.102 | No |
| ALT | 1.929 | 1 | 1.389 | No |
| BUN | 1.945 | 1 | 1.395 | No |
| LDL | 2.914 | 4 | 1.143 | No |
| Smoking | 1.181 | 1 | 1.087 | No |
| Previous stroke/TIA | 1.384 | 1 | 1.176 | No |
| Hypertension | 1.039 | 1 | 1.02 | No |
| DM | 1.176 | 1 | 1.085 | No |
| Hyperlipidemia | 1.167 | 1 | 1.08 | No |
| AF | 1.542 | 1 | 1.242 | No |
| CHD | 2.265 | 1 | 1.505 | No |
| Stroke etiology | 1.25 | 5 | 1.023 | No |
| NIHSS score at admission | 1.331 | 1 | 1.154 | No |

Note: GVIF, generalized variance inflation factor; Df, degrees of freedom.
