# Supplementary material for: The Prognostic Role of C‐Reactive Protein–Triglyceride Glucose Index in Predicting Unfavorable Outcomes in Acute Ischemic Stroke: A Large‐Scale Cohort Study
Source: Brain Behav. 2026 Jul 9;16(7):e71578. doi: 10.1002/brb3.71578 (PMC13347318; doi:10.1002/brb3.71578)
Supplement: Supplementary file 3 — Supplementary Table S3: brb371578‐sup‐0003‐TableS3.docx [file BRB3-16-e71578-s001.docx]

| Table S3 Baseline characteristics of included and excluded patients in the study cohort | | | | |
| --- | --- | --- | --- | --- |
| Characteristic | Total | Excluded | Included | *p* |
| Participants | 1906 | 421 | 1485 |  |
| Sex |  |  |  | 0.735 |
| Male | 1168 (61.28) | 255 (60.57) | 913 (61.48) |  |
| Female | 738 (38.72) | 166 (39.43) | 572 (38.52) |  |
| Age (years) |  |  |  | 0.147 |
| < 60 | 436 (22.88) | 112 (26.6) | 324 (21.82) |  |
| 60 to < 70 | 505 (26.50) | 112 (26.6) | 393 (26.46) |  |
| 70 to < 80 | 670 (35.15) | 141 (33.49) | 529 (35.62) |  |
| ≥ 80 | 295 (15.48) | 56 (13.3) | 239 (16.09) |  |
| BMI (kg/m2) | 23.50 ± 3.25 | 23.56 ± 3.17 | 23.48 ± 3.28 | 0.657 |
| Smoking, n (%) |  |  |  | 0.098 |
| No | 1156 (60.65) | 270 (64.13) | 886 (59.66) |  |
| Yes | 750 (39.35) | 151 (35.87) | 599 (40.34) |  |
| Hypertenion, n (%) |  |  |  | 0.865 |
| No | 695 (36.46) | 155 (36.82) | 540 (36.36) |  |
| Yes | 1211 (63.54) | 266 (63.18) | 945 (63.64) |  |
| Previous stroke/TlA, n (%) |  |  |  | 0.481 |
| No | 1504 (78.91) | 327 (77.67) | 1177 (79.26) |  |
| Yes | 402 (21.09) | 94 (22.33) | 308 (20.74) |  |
| CHD, n (%) |  |  |  | 0.535 |
| No | 1686 (88.46) | 376 (89.31) | 1310 (88.22) |  |
| Yes | 220 (11.54) | 45 (10.69) | 175 (11.78) |  |
| Hyperlipidemia, n (%) |  |  |  | 0.464 |
| No | 1207 (63.33) | 273 (64.85) | 934 (62.9) |  |
| Yes | 699 (36.67) | 148 (35.15) | 551 (37.1) |  |
| Atrial fibrillation, n (%) |  |  |  | 0.581 |
| No | 1499 (78.65) | 327 (77.67) | 1172 (78.92) |  |
| Yes | 407 (21.35) | 94 (22.33) | 313 (21.08) |  |
| Stroke etiology, n (%) |  |  |  | 0.161 |
| LAA | 606 (31.79) | 116 (27.55) | 490 (33) |  |
| SVO | 365 (19.15) | 82 (19.48) | 283 (19.06) |  |
| CE | 493 (25.87) | 119 (28.27) | 374 (25.19) |  |
| Other determined | 171 ( 8.97) | 47 (11.16) | 124 (8.35) |  |
| Undetermined | 270 (14.17) | 57 (13.54) | 213 (14.34) |  |
| mRS at admission, n (%) |  |  |  | 0.492 |
| 0 | 1389 (72.91) | 297 (70.55) | 1092 (73.58) |  |
| 1 | 176 ( 9.24) | 40 (9.5) | 136 (9.16) |  |
| 2 | 114 ( 5.98) | 32 (7.6) | 82 (5.53) |  |
| 3 | 99 ( 5.20) | 22 (5.23) | 77 (5.19) |  |
| 4 | 73 ( 3.83) | 20 (4.75) | 53 (3.57) |  |
| 5 | 54 ( 2.83) | 10 (2.38) | 44 (2.96) |  |
| Outcome |  |  |  | 0.164 |
| Favorable outcome | 1360 (71.35) | 289 (68.65) | 1071 (72.12) |  |
| Unfavorable outcome | 546 (28.65) | 132 (31.35) | 414 (27.88) |  |
| Laboratory parameters |  |  |  |  |
| WBC (10^9/L) | 8.14 ± 2.89 | 7.90 ± 2.59 | 8.20 ± 2.96 | 0.06 |
| HGB (g/dL) | 13.48 ± 2.00 | 13.40 ± 2.08 | 13.50 ± 1.98 | 0.356 |
| HCT (%) | 40.07 ± 5.59 | 39.73 ± 5.82 | 40.17 ± 5.52 | 0.152 |
| FIB (mg/L) | 330.33 ± 91.93 | 322.88 ± 97.72 | 332.44 ± 90.14 | 0.06 |
| PLT, Mean ± SD | 223.61 ± 71.31 | 218.07 ± 76.26 | 225.19 ± 69.79 | 0.071 |
| MCV, Mean ± SD | 92.96 ± 5.22 | 93.04 ± 5.06 | 92.93 ± 5.26 | 0.696 |
| Scr (mg/dl) | 0.89 (0.74, 1.08) | 0.91 (0.75, 1.10) | 0.88 (0.73, 1.08) | 0.199 |
| ALT (U/L) | 26.11 ± 14.34 | 26.61 ± 16.20 | 25.96 ± 13.77 | 0.412 |
| AST (U/L) | 18.00 (13.00, 26.00) | 18.00 (14.00, 25.00) | 18.00 (13.00, 26.00) | 0.649 |

Note: Variables are presented as mean ± SD, median (IQR) or n (%).

BMI, body mass index; NGR, normal glucose regulation; Pre-DM, prediabetes mellitus; DM, diabetes mellitus; TIA, transient ischemic attack; CHD, coronary heart disease; mRS, modified rankin scale; NIHSS, national institutes of health stroke scale; WBC, white blood cell; HGB, hemoglobin; HCT, hematocrit; FIB, fibrinogen; PLT, platelet; MCV, mean corpuscular volume; TG, triglyceride; TC, total cholesterol; HDL-C, high-density lipoprotein cholesterol; LDL-C, low-density lipoprotein cholesterol; BUN, blood urea nitrogen; Scr, serum creatinine; ALT, alanine aminotransferase; AST, aspartate aminotransferase; FBG, fasting blood glucose; hs-CRP, high-sensitivity C-reactive protein; CTI, c-reactive protein-triglyceride glucose index.
