# Supplementary material for: The Prognostic Role of C‐Reactive Protein–Triglyceride Glucose Index in Predicting Unfavorable Outcomes in Acute Ischemic Stroke: A Large‐Scale Cohort Study
Source: Brain Behav. 2026 Jul 9;16(7):e71578. doi: 10.1002/brb3.71578 (PMC13347318; doi:10.1002/brb3.71578)
Supplement: Supplementary file 4 — Supplementary Table S4: brb371578‐sup‐0004‐TableS4.docx [file BRB3-16-e71578-s003.docx]

| Table S4.  Baseline characteristics between participants favorable outcomes and unfavorable outcomes. | | | |
| --- | --- | --- | --- |
| **Variables** | **Favorable outcomes** | **Unfavorable outcomes** | ***p*** |
| Participants | 1071 | 414 |  |
| Sex |  |  | < 0.001 |
| Male | 704 (65.7) | 209 (50.5) |  |
| Female | 367 (34.3) | 205 (49.5) |  |
| Age (years) |  |  | < 0.001 |
| < 60 | 266 (24.8) | 58 (14) |  |
| 60 to < 70 | 307 (28.7) | 86 (20.8) |  |
| 70 to < 80 | 370 (34.5) | 159 (38.4) |  |
| ≥ 80 | 128 (12) | 111 (26.8) |  |
| BMI (kg/m^2^) | 23.7 ± 3.1 | 22.8 ± 3.5 | < 0.001 |
| Smoking, n (%) |  |  | < 0.001 |
| No | 603 (56.3) | 283 (68.4) |  |
| Yes | 468 (43.7) | 131 (31.6) |  |
| Hypertenion, n (%) |  |  | 0.026 |
| No | 408 (38.1) | 132 (31.9) |  |
| Yes | 663 (61.9) | 282 (68.1) |  |
| NGR, n (%) |  |  | 0.004 |
| No | 771 (72) | 328 (79.2) |  |
| Yes | 300 (28) | 86 (20.8) |  |
| Pre-DM, n (%) |  |  | 0.576 |
| No | 514 (48) | 192 (46.4) |  |
| Yes | 557 (52) | 222 (53.6) |  |
| DM, n (%) |  |  | 0.013 |
| No | 762 (71.1) | 267 (64.5) |  |
| Yes | 309 (28.9) | 147 (35.5) |  |
| Previous stroke/TlA, n (%) |  |  | < 0.001 |
| No | 883 (82.4) | 294 (71) |  |
| Yes | 188 (17.6) | 120 (29) |  |
| CHD, n (%) |  |  | 0.617 |
| No | 942 (88) | 368 (88.9) |  |
| Yes | 129 (12) | 46 (11.1) |  |
| Hyperlipidemia, n (%) |  |  | 0.061 |
| No | 658 (61.4) | 276 (66.7) |  |
| Yes | 413 (38.6) | 138 (33.3) |  |
| Atrial fibrillation, n (%) |  |  | < 0.001 |
| No | 884 (82.5) | 288 (69.6) |  |
| Yes | 187 (17.5) | 126 (30.4) |  |
| Stroke etiology, n (%) |  |  | < 0.001 |
| LAA | 362 (33.8) | 128 (31) |  |
| SVO | 236 (22) | 47 (11.4) |  |
| CE | 242 (22.6) | 132 (32) |  |
| Other determined | 70 (6.5) | 54 (13.1) |  |
| Undetermined | 161 (15) | 52 (12.6) |  |
| mRS at admission, n (%) |  |  | < 0.001 |
| 0 | 837 (78.2) | 255 (61.7) |  |
| 1 | 109 (10.2) | 27 (6.5) |  |
| 2 | 56 (5.2) | 26 (6.3) |  |
| 3 | 45 (4.2) | 32 (7.7) |  |
| 4 | 14 (1.3) | 39 (9.4) |  |
| 5 | 10 (0.9) | 34 (8.2) |  |
| NIHSS score at admission, n (%) |  |  | < 0.001 |
| ≤ 5 | 812 (75.8) | 107 (25.8) |  |
| 5 to ≤ 13 | 226 (21.1) | 200 (48.3) |  |
| > 13 | 33 (3.1) | 107 (25.8) |  |
| Laboratory parameters |  |  |  |
| WBC (10^9/L) | 8.0 ± 2.7 | 8.7 ± 3.5 | < 0.001 |
| HGB (g/dL) | 13.7 ± 1.9 | 12.9 ± 2.1 | < 0.001 |
| HCT (%) | 40.7 ± 5.2 | 38.7 ± 6.0 | < 0.001 |
| FIB (mg/L) | 325.9 ± 85.8 | 349.3 ± 98.5 | < 0.001 |
| PLT, Mean ± SD | 225.8 ± 66.5 | 223.6 ± 77.7 | 0.579 |
| MCV, Mean ± SD | 93.1 ± 5.0 | 92.6 ± 6.0 | 0.108 |
| TG (mg/dl) | 112.8 ± 56.1 | 102.2 ± 50.0 | < 0.001 |
| TC, Mean ± SD | 182.5 ± 43.7 | 175.5 ± 45.1 | 0.006 |
| HDL-C (mg/dl) | 46.8 ± 13.4 | 46.3 ± 14.2 | 0.568 |
| LDL-C (mg/dl) | 109.8 ± 37.6 | 106.2 ± 40.2 | 0.105 |
| BUN (mg/dl) | 17.3 ± 8.5 | 18.0 ± 9.8 | 0.151 |
| Scr (mg/dl) | 0.9 (0.8, 1.1) | 0.8 (0.7, 1.0) | 0.012 |
| ALT (U/L) | 19.0 (14.0, 26.0) | 17.0 (11.0, 25.8) | < 0.001 |
| AST (U/L) | 25.5 ± 13.7 | 27.1 ± 13.9 | 0.048 |
| ALB (g/dL) | 4.1 ± 0.4 | 3.9 ± 0.5 | < 0.001 |
| FBG (mg/dl) | 102.8 ± 32.6 | 116.4 ± 49.6 | < 0.001 |
| hs-CRP (mg/L) | 0.1 (0.1, 0.4) | 0.3 (0.1, 1.6) | < 0.001 |
| CTI | 3.8 ± 0.7 | 4.2 ± 0.9 | < 0.001 |
