# Supplementary material for: The Prognostic Role of C‐Reactive Protein–Triglyceride Glucose Index in Predicting Unfavorable Outcomes in Acute Ischemic Stroke: A Large‐Scale Cohort Study
Source: Brain Behav. 2026 Jul 9;16(7):e71578. doi: 10.1002/brb3.71578 (PMC13347318; doi:10.1002/brb3.71578)
Supplement: Supplementary file 5 — Supplementary Table S5: brb371578‐sup‐0005‐TableS5.docx [file BRB3-16-e71578-s008.docx]

| Tabel S5.  Associations of CRP and TyG index with the unfavorable outcomes of stroke. | | | | | | | | | | | | |
| --- | --- | --- | --- | --- | --- | --- | --- | --- | --- | --- | --- | --- |
| **Characteristic** | **Event, (n%)** | **Crude model** | |  | **Model 1** | |  | **Model 2** | |  | **Model 3** | |
|  |  | **OR (95%CI)** | ***p*** |  | **OR (95%CI)** | ***p*** |  | **OR (95%CI)** | ***p*** |  | **OR (95%CI)** | ***p*** |
| hs-CRP (per 1 unit) | 414 (27.9) | 1.142 (1.097–1.189) | <0.001 |  | 1.13 (1.08–1.17) | <0.001 |  | 1.09 (1.045–1.137) | 1e-04 |  | 1.06 (1.012–1.111) | 0.014 |
| hs-CRP |  |  |  |  |  |  |  |  |  |  |  |  |
| Q1 | 100 (20.2) | 1(Ref) |  |  | 1(Ref) |  |  | 1(Ref) |  |  | 1(Ref) |  |
| Q2 | 123 (24.8) | 1.555 (1.14–2.122) | 0.0054 |  | 1.55 (1.13–2.13) | 0.006 |  | 1.531 (1.111–2.108) | 0.009 |  | 1.216 (0.851–1.737) | 0.283 |
| Q3 | 191 (38.6) | 3.316 (2.472–4.448) | <0.001 |  | 3.2 (2.37–4.33) | <0.001 |  | 2.761 (2.005–3.803) | <0.001 |  | 1.643 (1.142–2.364) | 0.008 |
| P for trend |  | 1.849 (1.596–2.142) | <0.001 |  | 1.81 (1.56–2.11) | <0.001 |  | 1.669 (1.422–1.96) | <0.001 |  | 1.283 (1.069–1.54) | 0.007 |
| TyG (per 1 unit) | 414 (27.9) | 1.02  (0.84–1.25) | 0.826 |  | 1.16 (0.94–1.43) | 0.169 |  | 1.285 (1.023–1.614) | 0.031 |  | 1.086 (0.826–1.429) | 0.555 |
| TyG |  |  |  |  |  |  |  |  |  |  |  |  |
| Q1 | 144 (29.1) | 1(Ref) |  |  | 1(Ref) |  |  | 1(Ref) |  |  | 1(Ref) |  |
| Q2 | 130 (26.3) | 0.87  (0.66–1.15) | 0.33 |  | 0.94 (0.71–1.26) | 0.684 |  | 0.957 (0.711–1.289) | 0.773 |  | 0.715 (0.504–1.014) | 0.060 |
| Q3 | 140 (28.2) | 0.96  (0.73–1.26) | 0.763 |  | 1.09 (0.82–1.45) | 0.548 |  | 1.234 (0.912–1.671) | 0.173 |  | 0.952 (0.666–1.362) | 0.789 |
| P for trend |  | 0.98  (0.85–1.12) | 0.762 |  | 1.04 (0.9–1.21) | 0.552 |  | 1.11 (0.953–1.293) | 0.180 |  | 0.973 (0.811–1.166) | 0.764 |

Crude model: we did not adjust for other covariates;
Model 1: Age and sex;
Model 2: Age, sex, BMI, WBC, HGB, AST, ALT, BUN, and LDL;
Model 3: Age, sex, BMI, WBC, HGB, AST, ALT, BUN, LDL, smoking, previous stroke/TIA, hypertension, DM, hyperlipidemia, AF, CHD, stroke etiology, and NIHSS score at admission.
