# Supplementary material for: The Prognostic Role of C‐Reactive Protein–Triglyceride Glucose Index in Predicting Unfavorable Outcomes in Acute Ischemic Stroke: A Large‐Scale Cohort Study
Source: Brain Behav. 2026 Jul 9;16(7):e71578. doi: 10.1002/brb3.71578 (PMC13347318; doi:10.1002/brb3.71578)
Supplement: Supplementary file 6 — Supplementary Table S6: brb371578‐sup‐0006‐TableS6.docx [file BRB3-16-e71578-s004.docx]

| Tabel S6.  Association between CTI and unfavorable outcomes 3 months after stroke in female and male | | | | | | | | | | | | |  |
| --- | --- | --- | --- | --- | --- | --- | --- | --- | --- | --- | --- | --- | --- |
| **Sex** | **Characteristic** | **Event, (n%)** | **Crude model** | |  | **Model 1** | |  | **Model 2** | |  | **Model 3** | |
|  |  |  | **OR (95%CI)** | ***p*** |  | **OR (95%CI)** | ***p*** |  | **OR (95%CI)** | **p** |  | **OR (95%CI)** | ***p*** |
| **Male** | CTI (per 1 unit) | 209 (22.9) | 2.149 (1.736–2.659) | <0.001 |  | 2.13 (1.72–2.64) | <0.001 |  | 2.081 (1.638–2.645) | <0.001 |  | 1.591 (1.235–2.05) | <0.001 |
|  | CTI |  |  |  |  |  |  |  |  |  |  |  |  |
|  | Q1 | 42 (13.8) | 1(Ref) |  |  | 1(Ref) |  |  | 1(Ref) |  |  | 1(Ref) |  |
|  | Q2 | 60 (19.7) | 1.534 (0.997–2.361) | 0.052 |  | 1.53 (0.99–2.36) | 0.056 |  | 1.512 (0.972–2.352) | 0.067 |  | 1.32 (0.799–2.182) | 0.279 |
|  | Q3 | 107 (35.1) | 3.371 (2.256–5.038) | <0.001 |  | 3.41 (2.27–5.11) | <0.001 |  | 3.211 (2.088–4.936) | <0.001 |  | 2.309 (1.433–3.719) | <0.001 |
|  | *p* for trend |  | 1.874 (1.532–2.292) | <0.001 |  | 1.88 (1.54–2.31) | <0.001 |  | 1.817 (1.464–2.256) | <0.001 |  | 1.539 (1.212–1.953) | <0.001 |
| **Female** | CTI (per 1 unit) | 205 (35.8) | 1.892 (1.513–2.366) | <0.001 |  | 1.77 (1.41–2.23) | <0.001 |  | 1.653 (1.273–2.148) | <0.001 |  | 1.347 (1.004–1.807) | 0.047 |
|  | CTI |  |  |  |  |  |  |  |  |  |  |  |  |
|  | Q1 | 50 (26.2) | 1(Ref) |  |  | 1(Ref) |  |  | 1(Ref) |  |  | 1(Ref) |  |
|  | Q2 | 63 (33.2) | 1.399 (0.899–2.176) | 0.137 |  | 1.31 (0.82–2.07) | 0.256 |  | 1.355 (0.849–2.164) | 0.203 |  | 1.142 (0.66–1.976) | 0.635 |
|  | Q3 | 92 (48.2) | 2.621 (1.705–4.027) | <0.001 |  | 2.31 (1.48–3.62) | <0.001 |  | 2.086 (1.274–3.416) | 0.003 |  | 1.563 (0.882–2.771) | 0.126 |
|  | *p* for trend |  | 1.63 (1.313–2.022) | <0.001 |  | 1.53 (1.22–1.92) | <0.001 |  | 1.444 (1.128–1.849) | 0.004 |  | 1.251 (0.939–1.667) | 0.127 |

Crude model: we did not adjust for other covariates;
Model 1: Age;
Model 2: Age, BMI, WBC, HGB, AST, ALT, BUN, and LDL;
Model 3: Age, BMI, WBC, HGB, AST, ALT, BUN, LDL, smoking, previous stroke/TIA, hypertension, DM, hyperlipidemia, AF, CHD, stroke etiology, and NIHSS score at admission.
