# Supplementary figures and images for: The Prognostic Role of C‐Reactive Protein–Triglyceride Glucose Index in Predicting Unfavorable Outcomes in Acute Ischemic Stroke: A Large‐Scale Cohort Study
Source: Brain Behav. 2026 Jul 9;16(7):e71578. doi: 10.1002/brb3.71578 (PMC13347318; doi:10.1002/brb3.71578)

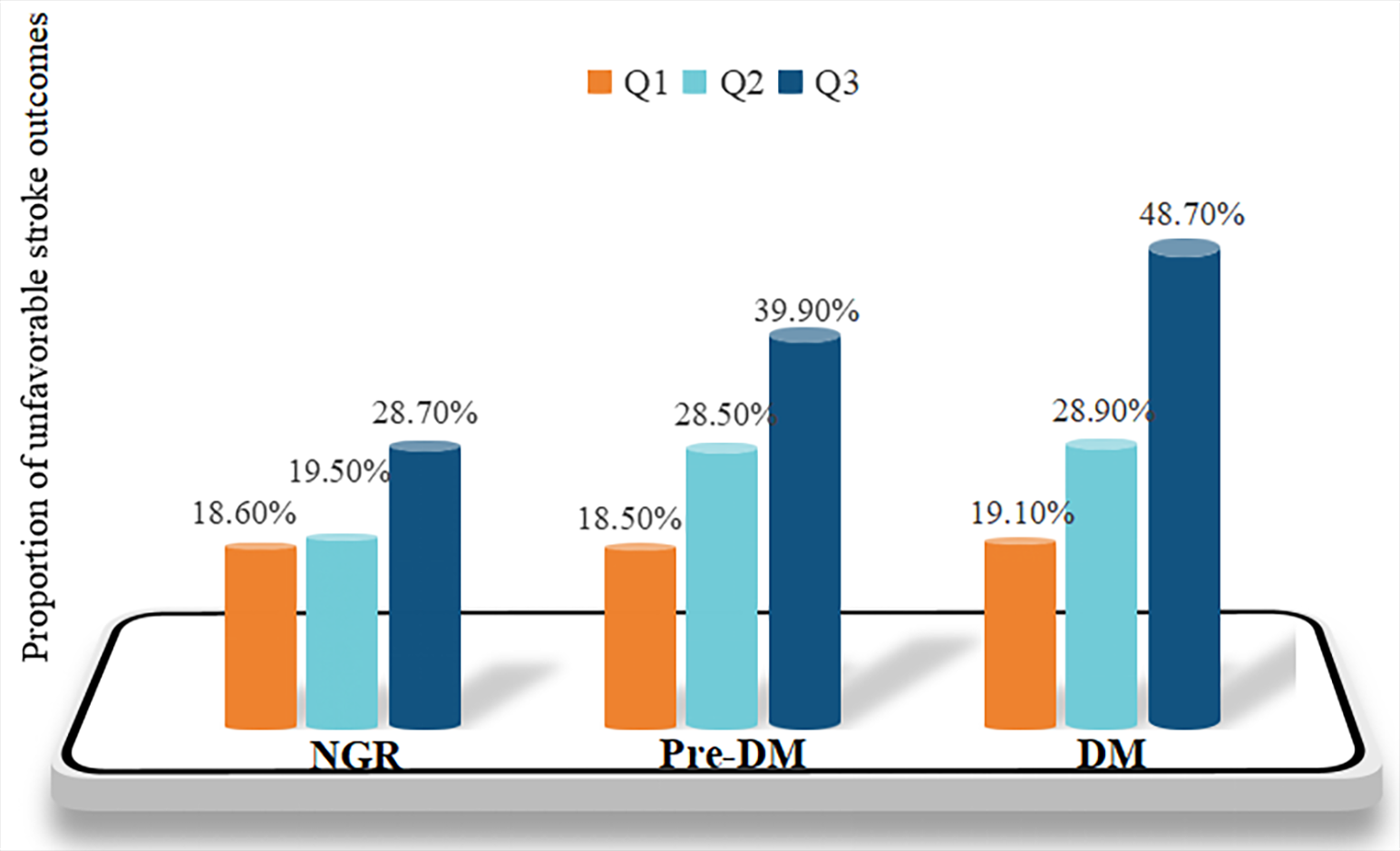


Fig. S1 Proportion of unfavorable outcomes in AIS by NGR, Pre-DM, and DM quartiles.

Supplement: Supplementary file 7 — Supplementary Figure S1: brb371578‐sup‐0007‐FigureS1.docx [file BRB3-16-e71578-s005.docx]
