# Supplementary material for: The Prognostic Role of C‐Reactive Protein–Triglyceride Glucose Index in Predicting Unfavorable Outcomes in Acute Ischemic Stroke: A Large‐Scale Cohort Study
Source: Brain Behav. 2026 Jul 9;16(7):e71578. doi: 10.1002/brb3.71578 (PMC13347318; doi:10.1002/brb3.71578)
Supplement: Supplementary file 8 — Supplementary Table S7: brb371578‐sup‐0008‐TableS7.docx [file BRB3-16-e71578-s009.docx]

| **Tabel S7. Association between CTI and unfavorable outcomes 3 months after stroke in different models excluding hypertension，hyperlipidemia, and CHD.** | | | | | | | | | | | | |
| --- | --- | --- | --- | --- | --- | --- | --- | --- | --- | --- | --- | --- |
| **Characteristic** | **Event, (n%)** | **Crude model** | |  | **Model 1** | |  | **Model 2** | |  | **Model 3** | |
|  |  | **OR (95%CI)** | ***p*** |  | **OR (95%CI)** | ***p*** |  | **OR (95%CI)** | ***p*** |  | **OR (95%CI)** | ***p*** |
| CTI (per 1 unit) | 93 (26.1) | 2.181 (1.585-3.002) | <0.001 |  | 2.25 (1.62-3.13) | <0.001 |  | 2.015 (1.398-2.904) | <0.001 |  | 1.673 (1.131-2.474) | 0.01 |
| CTI |  |  |  |  |  |  |  |  |  |  |  |  |
| Q1 (<3.55) | 21 (17.6) | 1(Ref) |  |  | 1(Ref) |  |  | 1(Ref) |  |  | 1(Ref) |  |
| Q2 (3.55-4.20) | 26 (22) | 1.319 (0.694-2.505) | 0.398 |  | 1.28 (0.67-2.46) | 0.459 |  | 1.31 (0.662-2.593) | 0.438 |  | 1.508 (0.694-3.276) | 0.3 |
| Q3 (>4.20) | 46 (38.7) | 2.941 (1.616-5.35) | <0.001 |  | 3.04 (1.64-5.63) | <0.001 |  | 2.576 (1.322-5.02) | 0.006 |  | 2.219 (1.044-4.72) | 0.038 |
| *p* for trend | 93 (26.1) | 1.754 (1.295-2.375) | <0.001 |  | 1.79 (1.31-2.44) | <0.001 |  | 1.629 (1.164-2.279) | 0.005 |  | 1.489 (1.023-2.166) | 0.038 |

Crude model: we did not adjust for other covariates;
Model 1: Age;
Model 2: Age, BMI, WBC, HGB, AST, ALT, BUN, and LDL;
Model 3: Age, BMI, WBC, HGB, AST, ALT, BUN, LDL, smoking, previous stroke/TIA, hypertension, hyperlipidemia, AF, CHD, stroke etiology, and NIHSS score at admission.
