# Supplementary material for: The Prognostic Role of C‐Reactive Protein–Triglyceride Glucose Index in Predicting Unfavorable Outcomes in Acute Ischemic Stroke: A Large‐Scale Cohort Study
Source: Brain Behav. 2026 Jul 9;16(7):e71578. doi: 10.1002/brb3.71578 (PMC13347318; doi:10.1002/brb3.71578)
Supplement: Supplementary file 9 — Supplementary Figure S2: brb371578‐sup‐0009‐FigureS2.docx [file BRB3-16-e71578-s007.docx]

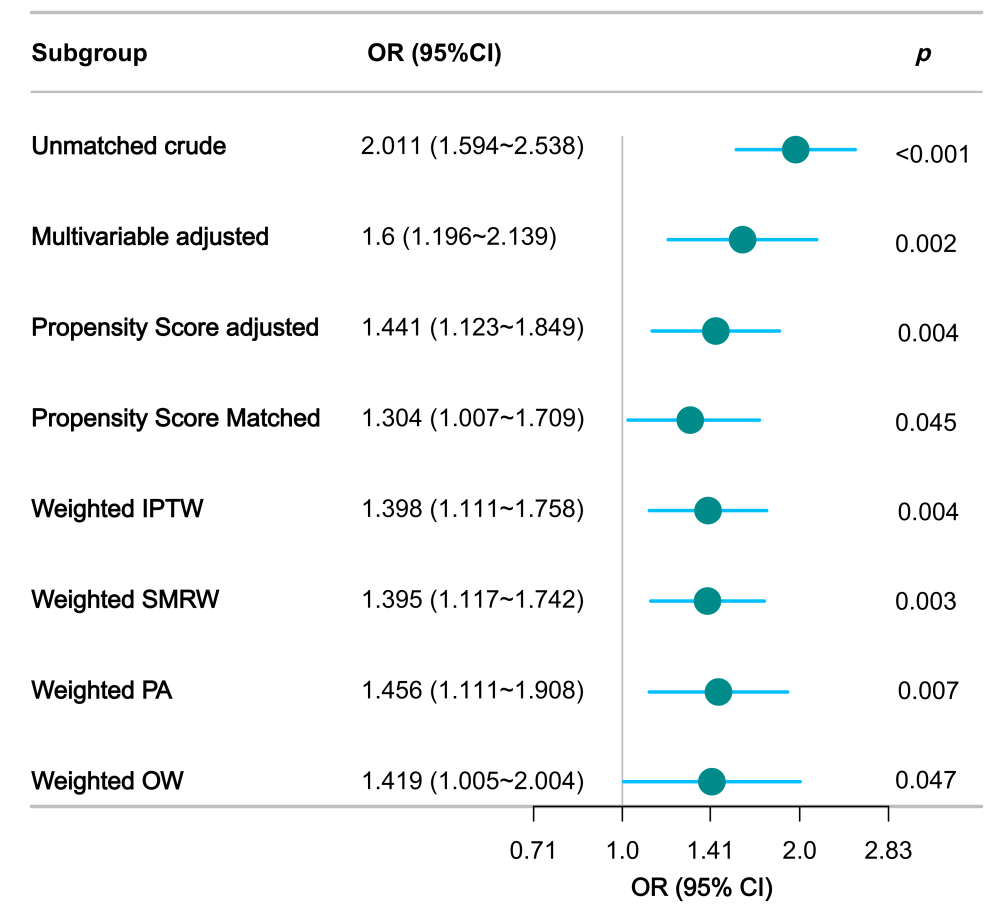


Fig. S2 Forest plot of propensity score analysis for CTI and AIS unfavorable outcomes. Adjusted for age, sex, BMI, WBC, HGB, AST, ALT, BUN, LDL,smoking, previous stroke/TIA, hypertension, DM, hyperlipidemia, AF,CHD, stroke etiology, and NIHSS score at admission.
